# An Improved Real-Time Viability PCR Assay to Detect *Salmonella* in a Culture-Independent Era

**DOI:** 10.3390/ijms232314708

**Published:** 2022-11-25

**Authors:** Surangi H. Thilakarathna, Taryn Stokowski, Linda Chui

**Affiliations:** 1 Department of Laboratory Medicine and Pathology, University of Alberta, Edmonton, AB T6G 1C9, Canada; 2 Alberta Precision Laboratories, Public Health Laboratory (ProvLab), Edmonton, AB T6G 2J2, Canada

**Keywords:** *Salmonella*, viability PCR, photoactivation, selective DNA amplification, propidium monoazide, PMAxx™, false positive, false negative, culture-independent diagnostics

## Abstract

Viability PCR (vPCR) uses a DNA intercalating dye to irreversibly bind double-stranded DNA from organisms with compromised cell membranes. This allows the selective amplification of DNA from intact cells. An optimized vPCR protocol should minimize false positives (DNA from compromised cells not fully removed) and false negatives (live cell DNA bound by the dye). We aimed to optimize a vPCR protocol using PMAxx™ as the intercalating agent and *Salmonella* Enteritidis as the target organism. To do this, we studied (1) single vs. sequential PMAxx™ addition; (2) a wash step post-PMAxx™ treatment; (3) a change of tube post-treatment before DNA extraction. The single vs. sequential PMAxx™ addition showed no difference. Results signified that PMAxx™ potentially attached to polypropylene tube walls and bound the released DNA from PMA-treated live cells when lysed in the same tube. A wash step was ineffective but transfer of the treated live cells to a new tube minimized these false-negative results. Our optimized protocol eliminated 10^8^ CFU/mL heat-killed cell DNA in the presence of different live cell dilutions without compromising the amplification of the live cells, minimizing false positives. With further improvements, vPCR has great potential as a culture-independent diagnostic tool.

## 1. Introduction

Real-time quantitative PCR (qPCR) is a molecular technique that is widely used in microbiological research and the clinical diagnostic setting. Using a high-volume, multi-analyte amplification platform, qPCR can identify multiple pathogens in a single clinical sample. The assay can provide high sensitivity, rapid turnaround time, and is less labor-intensive through automation [1,2]. However, qPCR is limited by its inability to distinguish between the DNA from live versus dead cells. This major limitation associated with qPCR can be addressed by viability PCR (vPCR), where qPCR signals from dead cell DNA are removed by a viability dye [3]. A viability dye such as propidium monoazide (PMA) can penetrate through cells with compromised membranes, such as dead or damaged cells, and irreversibly bind to the DNA upon exposure to intense visible light [4]. This irreversible binding prevents DNA amplification such that no qPCR signal will be generated from the dead cells. The intact live cell membranes are impermeable to the viability dye, and therefore, in a mixture of live and dead cells, only live cell DNA will be selectively amplified in a qPCR assay.

Current vPCR assays that use PMA involve a three-step process: addition of PMA to a cell suspension (live and/or dead cells), followed by an incubation period in the dark, and then photoactivation of PMA by exposure to intense visible light. After this treatment, cells are lysed to release the DNA for qPCR amplification. Ideally, as mentioned above, qPCR signals should be generated from only live cell DNA; however, there are pitfalls associated with current vPCR assays that lead to false-positive and false-negative results [5,6]. False positives are generated when the viability dye is incapable of fully removing the dead cell DNA and thereby, result in an overestimation of the initial load of live microbes. Conversely, false negatives have been associated with dye binding to live cell DNA [7]. Consequently, the uncertainty of these assays significantly hinders the applicability of vPCR as a diagnostic tool.

Numerous approaches have been suggested by different investigators to improve the performance of vPCR assays such as (1) using better-performing viability dyes that are fully impermeant to intact cell membranes [7]; (2) combining mixtures of dyes that target dead cells with both compromised cells membranes, as well as intact cell membranes, i.e., ghost cells) [8]; (3) applying an appropriate dye concentration that completely removes dead cell DNA [9], and also sequential PMA treatments [5,6,10]. Other suggested improvements also include adjusting the length of the photoactivation period [11], as well as the number of photoactivation applications [11,12], and selecting an optimal amplicon length that shows an increased cycle threshold difference between live and dead bacterial cells without overly compromising the qPCR efficiency [13]. Despite all the efforts, the issues of false-positive/negative results have not been resolved to date.

According to the Public Health Agency of Canada, *Salmonella* is the most common gastroenteric pathogen reported to the National Enteric Surveillance program [14], and each year, 1 in 4 hospitalizations related to food-related illnesses was due to salmonellosis [15]. Among the isolated serotypes reported, *Salmonella. enterica* subspecies (subsp.) *enterica* serotype (ser.) Enteritidis (*S*. Enteritidis) was the most frequent [16]. We used *S.* Enteritidis as the model organism for our vPCR optimization study since this is the top *Salmonella* serotype causing acute gastroenteritis [14] in Canada. The *invA* gene is a highly conserved gene in the *Salmonella* genome that plays a crucial role in host epithelial cell invasion [17]. It is encoded in the *Salmonella* pathogenicity island-1 gene cluster and is detected in almost all *Salmonella* serotypes [17]. 

We aimed to optimize the vPCR protocol by minimizing false-positive and false-negative qPCR readings and using the recently developed PMAxx™ as the viability dye. Ethidium monoazide (EMA), another viability dye, is known to permeate live cells generating false negatives [7,18] and, therefore, was not considered for this study. Our main objectives were to minimize false qPCR positives by finding the optimum PMAxx™ concentration that can completely remove dead cell DNA and minimize false qPCR negatives by finding approaches, i.e., a wash step and a tube change after photoactivation, to remove the negative effects of PMAxx™ on live cells.

## 2. Results 

### 2.1. Sensitivity and Specificity of the In-House Primers and Probe

The sequences of the in-house primers and probe set can be found in Kellner et al. [19] and are also listed in the Supplementary Materials (Supplementary Table S1). A standard curve generated using ten-fold dilutions of *S.* Enteritidis was used to study the correlation between Ct values and plate counts (Supplementary Figure S1). The limit of detection (LOD) of the in-house primers and probe was at 10^3^ colony forming units (CFU)/mL, and the Ct cut-off value of the assay was 31 (Table 1). The amplification efficiency (E = −1 + 10 (−1/slope)) was 101.4%, which indicated that the primer–probe set was highly efficient. 

Similar results were obtained with the other 13 *Salmonella* serotypes (Supplementary Table S2); a standard curve generated based on the average Ct values from 2 replicates for the 13 serotypes had Y= −3.35X + 40.7 and R^2^ = 0.9964. The non-*Salmonella* strains in the exclusivity panel (Supplementary Table S2) tested negative, confirming the absence of cross-reactivity of the primers and probe used. 

### 2.2. Optimized PMAxx™ Treatment Conditions 

#### 2.2.1. Optimum PMAxx™ Concentration Required to Bind Heat-Killed Cell DNA

Heat-killed (HK) cells were prepared from *S.* Enteritidis, as explained in Section 4.5. Two different PMAxx™ concentrations (50 and 100 μM) were tested on 10^8^–10^5^ CFU/mL HK cells (Table 2), and the 100 μM PMAxx™ was applied as either a single (one dose of 100 μM) or a double application (2 doses of 50 μM). Fifty μM PMAxx™ was sufficient to completely bind DNA from 10^7^ CFU/mL HK cells but not 10^8^ CFU/mL HK cells. A 100 μM PMAxx™ was sufficient to bind to DNA from 10^8^ HK cells and resulted in a negative qPCR, and there was no difference between the single or sequential applications. Our results indicated that the total amount of PMAxx™ applied was more crucial than how it was administered (single or sequential) to achieve the optimum PMAxx™ performance. Therefore, a total concentration of 100 μM PMAxx™ was selected for all further experiments. All PMAxx™ treatments included 20% of DMSO, and the qPCR results showed improved PMAxx™ permeability in HK cells (i.e., higher Cts) but no effect on treated live cells.

#### 2.2.2. PMAxx™ Treatment on Live Cells

Since clinical and food samples and specimens consist of both live and dead cells, it was essential to study the effect of PMAxx™ treatment on live cells. Therefore, the 100 μM total PMAxx™ concentration was tested on different live cell dilutions. When PMAxx™-treated live cells were photoactivated and lysed in the same tube, there was a noticeable increase in the Ct values compared to the untreated live cells, especially when the cell concentration was at 10^6^ CFU/mL and below (Table 3). 

This result clearly demonstrated that PMAxx™ could affect the detection of the live cells when photoactivation and lysis were conducted in the same tube. This can result in an underestimation of the actual live cells in the sample; the increased Ct values can cause a false-negative qPCR result. The additional washing step after the PMAxx™ treatment did not improve the qPCR results. However, when lysis was performed in a new tube after PMAxx™ treatment and photoactivation, the Ct values were remarkably improved (Table 3). The delta Ct values between untreated and PMAxx™ treated (with tube change) still existed but were minimal. The LOD for live cells decreased by 1 log in the presence of PMAxx™ treatment (from 10^3^ CFU/mL to 10^4^ CFU/mL). 

#### 2.2.3. PMAxx™ Binding to Polypropylene Tube Walls

Results from the effect of PMAxx™ on live cells (Section 2.2.2) pointed towards the possibility of this specific dye attaching to polypropylene tube walls. To test this, tubes were pre-treated with 100 μM final concentration of PMAxx™ to coat the tube walls, and PMAxx™ was removed from the tubes with and without a washing step; 10^5^ CFU/mL HK cells in phosphate saline buffer (PBS) were added to the respective tubes and photoactivated before the lysis step for DNA extraction. Only HK cells were used for this proof-of-concept experiment. We observed a 5 Ct value increase (Ct = 30.79 ± 0.41) using DNA extracted from HK cells in photoactivated PMAxx™ pre-treated tubes as compared to untreated HK (Ct = 25.31 ± 0.3; *p* < 0.05). These results were mirrored in the set of PMAxx™-coated tubes that received a wash treatment before the cells were lysed (Ct = 30.12 ± 0.46). 

From these findings, there is evidence to show that PMAxx™ was attached to the tube walls (without being removed by washing) and remained active for a period of time after photoactivation. Consequently, the residual PMAxx™ on tube walls can bind to live cell DNA after lysis, leading to a significant Ct increase (compared to untreated live cells) when photoactivation and lysis were performed in the same tube (Table 3). Since attached PMAxx™ was left in the first tube, the effect of PMAxx™ was minimal on live cells when a second tube was used for lysis (Table 3). 

### 2.3. Optimized PMA Treatment Protocol on HK Cells and Live Cells Spiked with HK Cells

The optimized PMAxx™ treatment protocol consisted of a 100 μM total PMAxx™ concentration, a tube change after photoactivation, and PMAxx™ removal prior to tube change. Here, the optimized protocol was tested on HK cells. The 100 μM total PMAxx™ concentration eliminated the detection of 10^8^ CFU/mL HK cells (Table 4), signifying its effectiveness at higher HK cell concentrations. There was a Ct increase when untreated HK cells underwent a tube change suggesting that some of the cells attached to the wall of the original tube (Table 4). Thus, the tube change step in the optimized PMAxx™ treatment protocol further improved the HK cell removal. Tube change without the PMAxx™ treatment did not affect the Ct values of live cells (Table 3), proving that live cells with intact membranes do not readily attach to tube walls. According to Table 4, 10^4^ CFU/mL HK cells were completely eliminated during the tube change. It is difficult to approximate the average amount of cells removed due to the attachment as the Ct differences were inconsistent at different cell dilutions. 

**Figure 1:**
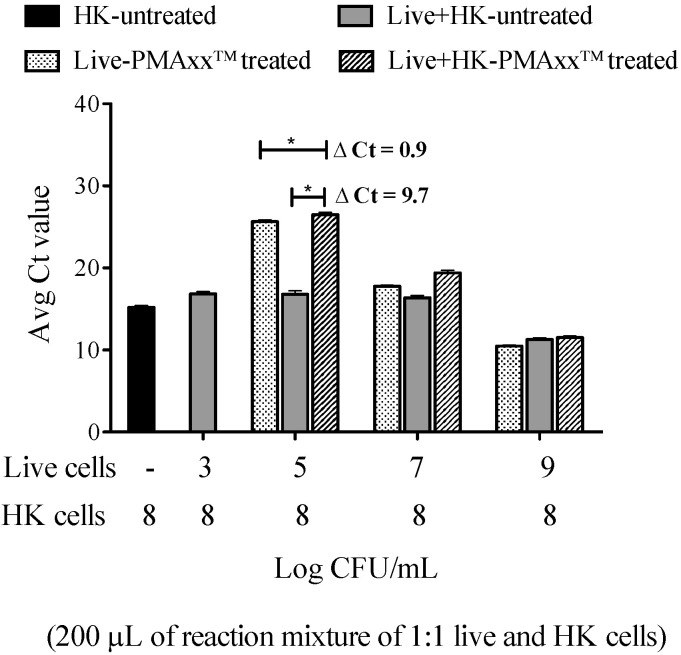
Effect of optimized PMAxx™ treatment on 10^8^ CFU/mL HK cells spiked into live cell dilutions from 10^9^ to 10^3^ CFU/mL. The total reaction volume for the live and HK cell mixture was 200 μL. Data were from 3 independent assays with 3 PCR replicates. HK-untreated and Live- PMAxx™ treated were used as treatment controls for Ct value comparisons of the cell mixtures. PCR results of 10^8^ CFU/mL HK- PMAxx™ treated, 10^3^ CFU/mL live- PMAxx™ treated, and 10^3^ CFU/mL live + 10^8^ CFU/mL HK- PMAxx™ treated were below the Ct cut-off value and were undetected and hence, not shown in the figure. ΔCt: difference in the cycle threshold. * denotes statistical significance (*p* < 0.05).

The optimized PMAxx™ treatment was then tested on different dilutions of live cells spiked with 10^8^ CFU/mL HK cells (Figure 1 and Supplementary Table S3). The Ct values for ‘live+HK untreated’ reflect both live and HK cell concentrations while the Ct values for ‘live+HK- PMAxx™ treated’ reflect the concentration of only live cells when the HK cells were removed by the PMAxx™ treatment. ‘Live+HK- PMAxx™-treated’ showed significantly higher Ct values compared to ‘live+HK untreated’ when live cell counts were less than 10^8^ CFU/mL HK cells denoting HK cell DNA was removed by the PMAxx™ treatment (*p* < 0.05). The close similarity between the Ct values for ‘live- PMAxx™-treated’ and ‘live+HK- PMAxx™ treated implied that the PMAxx™ treatment completely removed 10^8^ CFU/mL HK cell signals and selectively amplified the live cells in the live and HK cell mixture in pure culture.

## 3. Discussion

The main aim of this work was to explore strategies to overcome false positives and false negatives generated by current vPCR assays. Since its initial development [7], various modifications were suggested to overcome these limitations; however, they have not been fully resolved to date. In this study, we investigated a few simple yet effective vPCR modifications that can further minimize false-positive and false-negative results. 

EMA, also a phenanthridine compound, was used in vPCR developments prior to the use of PMA [3]. Presently, PMA is considered superior to EMA due to its superior impermeability to intact cell membranes hence being ‘less toxic’ to live cells [6,7]. Therefore, it is commonly used in vPCR protocols today. Biotium Inc. has recently introduced the next-generation vPCR dye, ‘PMAxx™’, which is a proprietary formulation and an improved version of PMA and claims to offer even better performance (Biotium Inc., Fremont, CA, USA; Product information sheet). Upon light exposure, PMA is converted to a highly reactive nitrene compound that irreversibly binds to double-stranded DNA [7]. This binding prevents DNA from amplifying and was believed to make the PMA-bound DNA water insoluble [4,6,7]. Once photoactivated and bound to DNA or other molecules such as water, PMA is expected to be inactive [7]. So, interference from residual photoactivated PMA on lysed live cell DNA was not anticipated [7]. However, our work clearly shows PMA can bind to DNA beyond the duration of photoactivation (Table 3). 

We used PMAxx™, the improved version of PMA (Biotium Inc. Fremont, CA, USA), in our vPCR experiments. We prepared the working PMAxx™ solution by diluting the 2 mM PMAxx™ stock solution in 20% DMSO and DNA/RNA-free water. DMSO has been used in previous studies to enhance PMA activity [12,20,21] without negatively affecting the membrane permeability of live cells. It was crucial for us to select the optimum PMAxx™ concentration that effectively removed higher HK cell counts. In our study, a final concentration of 100 μM PMAxx™ effectively removed dead cell DNA from 10^8^ CFU/mL HK cells. Furthermore, the total amount of PMAxx™ was more critical than how it was administered: as a single treatment or as a sequential treatment. Previous studies have shown that sequential PMA treatments were more effective compared to single treatments on *Mycobacterium* [12] and *Escherichia coli* [22]. However, both studies compared PMA treatments added once vs. twice, i.e., 25 μM once (25 μM×1) vs. 25 μM twice (25 μM×2), without controlling for the overall concentration used. Therefore, the effects observed in those studies would likely be due to the increased amount of PMA added in the sequential treatment (total PMA) rather than its application as a single vs. sequential treatment.

In general, concentrations of 50–100 μM of PMA have been used in previous vPCR studies but those PMA concentrations did not completely remove the DNA from higher counts of dead cells, i.e., >10^6^ CFU/mL [12,21,23,24]. We achieved complete removal of 10^8^ CFU/mL HK cell signals with a 100 μM PMAxx™ treatment and a tube change step (Table 4). Although our main objective of using the tube change approach was to eliminate the effect of residual PMAxx™ on live cells, this step resulted in added benefits by being effective at removing HK cells from the cell suspension (Table 3 and Table 4). Previous studies have also used the tube change approach but to remove dead cells and extracellular DNA from cell suspensions [5,10,25]. The authors reported that part of the DNA from dead cells was not accessible to the viability dye as those dead cells were attached to the polypropylene tube walls. The DNA from these attached dead cells was released during the cell lysis step and contributed to a false-positive PCR reading [26]. To overcome this problem and remove more dead cells and extracellular DNA, the authors suggested multiple tube changes post-photoactivation [10,25]. Our results showed that ~10^4^ CFU/mL HK cells can be removed from a cell suspension by simply transferring them to a new tube. This bacterial cell adhesion, which is separate from the biological adhesion response, is linked to the surface charge of the bacterial cells [27]. Both Gram-negative and Gram-positive live bacterial cells hold an inherent negative surface charge or “zeta potential” that can get “less negative” due to heat treatments and increased membrane permeability [27,28]. This change in the zeta potential can promote adhesion to surfaces with a “more negative” charge, such as polypropylene surfaces that have a low wettability (e.g., a hydrophobic surface with a negative surface charge). This can be a possible explanation as to why attachment was observed for untreated HK cells but not untreated live cells (Table 3 and Table 4). 

Although higher PMAxx™ concentrations are effective at removing high HK cell concentrations of DNA, the use of higher PMAxx™ concentrations, i.e., 100 μM, was challenged with potential unfavorable effects on live cells. One major disadvantage was the partial removal of live cell signals causing false-negative qPCR results [12,23]. Our observations were similar, as the sensitivity of the qPCR assay was reduced from 10^3^ CFU/mL to 10^6^ CFU/mL when live cells were treated with PMAxx™ without a tube change (Table 3). Although the sensitivity improved with the tube change, the LOD was still reduced from 10^3^ CFU/mL to 10^4^ CFU/mL denoting false negatives at a lower live cell dilution. How PMA affects live cells is still unclear and its reactivity under different conditions is not completely understood. With our findings, we believe that PMA has a high tendency to attach to negatively charged polypropylene surfaces. Furthermore, contrary to the belief that PMA can be completely inactivated during the photoactivation step, our findings are evidence that PMAxx™ remained active and bound to DNA even after the 15 min photoactivation was complete. Therefore, it is possible that residual PMAxx™ attached to tube walls was capable of binding some of the released live cell DNA after lysis and leads to false-negative qPCR signals when photoactivation and lysis were performed in the same tube. When a new tube was used for lysis, the residual PMAxx™ effect was greatly reduced. To our understanding, the tube change approach to minimize PMA’s effect on live cells has not been reported in previous works. 

We observed that a tube change minimally affected live cells (Ct difference < 0.5 Ct), and the PMAxx™ treatment itself seemed to affect the live cells at lower concentrations. One possible explanation is that the carryover residual PMAxx™ transferred to the second tube and bound the live cell DNA after lysis in the second tube. Secondly, it is possible that PMAxx™ treatment altered the surface charge in some of the live cells with compromised cell membrane properties, allowing the live cells to attach to the tube wall. Therefore, when the live cells were transferred to a new tube for lysis, some of the cells were left in the first tube, leading to a higher Ct compared to untreated live cells. Thirdly, as suggested by previous authors, it is possible that PMA penetrated the live cells with damaged cell membranes and bound the DNA, preventing amplification [29]. One or all of these possibilities could have contributed to the observed effect of PMAxx™ on live cells and further investigations are warranted to gain a better understanding of how PMAxx™ affects live cells. 

We acknowledge the limitation of our protocol as false negatives were not eliminated at lower live cell dilutions. Further, as a general limitation with using any viability dye that uses cell membrane integrity as the viability criterion, dead cells with intact cell membranes, i.e., ghost cells, or cells inactivated by ultraviolet radiation (cells inactivated but do not lose the cell membrane integrity) will not be removed, leading to an overestimation of live/intact cells. However, this is less common in clinical settings. Even with these limitations, an improved vPCR assay can have practical applications as a viability assessment tool in different settings, such as in food safety environments and clinical laboratories. At present, culture-based methods are predominantly used for viability assessment and the slow turnaround time associated with these methods is a major limitation. Furthermore, vPCR assays can serve as a potential solution to assessing the viability of bacteria under challenging situations where culture-based methods are not an option, such as viable but not culturable bacteria [30] or slow-growing bacteria [12]. Our proposed vPCR protocol has addressed some of the major limitations associated with the current vPCR assays. Possible PMA attachment to polypropylene tube walls is a major finding in this study, and we believe this activity of PMA will be helpful for future vPCR assay development involving PMA. We believe our findings overall will address some of the nuances associated with vPCR assays. 

## 4. Materials and Methods

### 4.1. Bacterial Strains and Growth Conditions

All bacteria strains in the inclusivity and exclusivity panels were obtained from the Alberta Precision Laboratories: Alberta Public Health Laboratory (ProvLab) Quality Control Department (Edmonton, Alberta, Canada). These isolates were retrieved from frozen skim milk stored at −80 °C and cultured on sheep blood agar plates (BAP) (Dalynn Biologicals, Calgary, Alberta, Canada) and incubated at 37 °C overnight to use in experiments below.

For experiments involving a dilution series of *Salmonella*, a single colony was picked from the BAP the following day, inoculated into 4 mL of Trypticase Soy Broth (TSB) (Dalynn Biologicals, Calgary, AB, Canada), and incubated at 37 °C with moderate shaking (Model 4365, Thermo Fisher Scientific, Oakwood, OH, USA) for ~3.5 h. The suspension was then standardized to a reading of ~0.5 using a turbidity meter (MicroScan Turbidity Meter, Siemens Healthcare Diagnostics Ltd., Los Angeles, CA, USA). This standardized suspension was serially diluted and used for nucleic acid extraction as below. 

### 4.2. Nucleic Acid Extraction 

A 100 μL aliquot from each dilution was centrifuged at 17,115× *g* for 5 min. The supernatant was removed, and the cell pellets were resuspended in 100 μL of rapid lysis buffer (100 mM NaCl, 10 mM Tris-HCL pH 8.3, 1 mM EDTA pH 9.0, 1% Triton X-100), boiled at 95 °C for 15 min. The suspension was centrifuged at 17,115× *g* for 5 min, and 5 μL of the supernatant was used as the template for qPCR.

### 4.3. Primers, Probe, and qPCR Assay Conditions

A previously developed in-house primer–probe set was used for the TaqMan-chemistry-based qPCR assay [19]. The primers and probe (Integrated DNA Technologies, IDT, Skokie, IL, USA) targeted a region from the conserved *Salmonella invA* gene as shown in Supplementary Table S1. The qPCR reaction mixture was prepared by combining 10 μL of 2X PrimeTime^®^ Gene Expression Master mix (Integrated DNA Technologies, IDT, Skokie, IL, USA), 2 μL of nuclease-free water (Invitrogen™, Life Technologies, Grand Island, NY, USA), 3 μL of the in-house primer–probe mixture (0.22 μM final concentration of the probe, 0.33 μM final concentration of each of the primers), and 5 μL of the DNA template to contain a final reaction volume of 20 μL. Positive DNA for the *invA* gene and a no-template control (nuclease-free water) were included in each run. qPCR runs for each experiment were performed in triplicate, and each dilution per treatment was plated in triplicate qPCR wells unless otherwise stated. Amplification conditions consisted of 95 °C for 3 min, followed by 40 cycles of 95 °C for 5 s and 60 °C for 30 s on the 7500 FAST real-time PCR system (Applied Biosystems, Foster City, CA, USA). 

### 4.4. Sensitivity and Specificity Assays

#### 4.4.1. Inclusivity of the Primers and Probe and the Limit of Detection for the qPCR Assay

A clinical *S.* Enteritidis isolate, along with 13 other *Salmonella* serotypes (Supplementary Table S2), was cultured on BAP and incubated at 37 °C overnight. For the inclusivity assay, a colony qPCR was performed using the following method: a single colony was picked and lysed in 100 μL rapid lysis buffer as described in Section 4.2 for qPCR. Extracted DNA from 2 clinical strains, *S.* Enteritidis and *S.* Typhimurium, were used as positive controls, and a no-template control was included in each run. Experiments were repeated on 2 separate days and in triplicate wells per qPCR run.

After inclusivity was confirmed, the sensitivity of the primers and the probe was determined. A TSB culture was prepared as in Section 4.1, and serial 10-fold dilutions of each of the standardized cell suspensions were made from neat to 10^−8^ in PBS. One hundred microliter aliquots from dilutions 10^−6^ to 10^−8^ were inoculated onto BAP in triplicates and incubated overnight to determine the number of colony-forming units/mL (CFU/mL). DNA was extracted from 100 μL of each cell suspension as described above in Section 4.2, and 5 μL was used as a template for the qPCR assay. A standard curve was generated by correlating the CFU/mL and qPCR cycle threshold (Ct) values of all 14 isolates. The LOD was determined by the lowest bacterial cell dilution that was detected at 95% in all qPCR runs. This was the Ct cut-off value for our qPCR assay. 

#### 4.4.2. Exclusivity Assays

A panel of 17 common and rare non-*Salmonella* bacterial strains (Supplementary Table S2) found in Alberta was included in the exclusivity panel. Each bacterial strain was grown on a BAP overnight, and colony qPCR was performed as in Section 4.4.1. Extracted DNA from 2 clinical strains, *S.* Enteritidis and *S.* Typhimurium, were used as positive controls and a no-template control was included in each run. Experiments were repeated on 2 separate days and in triplicate wells per qPCR run.

### 4.5. Preparation of Live and Heat-Killed Cells

*Salmonella* Enteritidis in TSB culture was freshly prepared as mentioned in Section 4.4.1. One milliliter of broth culture was subjected to centrifugation at 17,115 × *g* for 5 min, and the harvested cell pellet was resuspended in PBS. A ten-fold serial dilution of live cells was prepared and divided into 2 sets. One set served as the live cell dilutions and a 100 μL aliquot of 10^−7^ and 10^−8^ were plated on BAP in triplicates for bacterial enumeration. The other set was heated at 95 °C for 5 min in a water bath to prepare the HK cells. The loss of viability of HK cells was confirmed by plating 100 μL of neat and 10^−1^, 10^−2^ dilutions on BAP. These sets of live and HK cells were used in the experiments below. 

### 4.6. Optimizing the PMAxx™ Treatment Conditions

#### 4.6.1. Optimum PMAxx™ Concentration to Remove HK Cell DNA

PMAxx™ is an improved proprietary version of PMA (Biotium Inc. Fremont, CA, USA). 20 mM PMAxx™ solution in water [Biotium Inc. Fremont, CA, USA] was used to prepare a 2 mM PMAxx™ stock solution containing 20% dimethylsulfoxide (DMSO, Sigma-Aldrich, St. Louis, MO, USA) in sterile DNA/RNA-free water (Invitrogen™, Life Technologies, Grand Island, NY, USA) which was stored at −20 °C in the dark until used. To preserve PMAxx™ ability to be activated by light, experiments were performed under minimal lighting conditions. 

PMAxx™ solution was added to serially diluted HK cell suspensions in PBS from neat to 10^−8^ as a single or a sequential PMAxx™ treatment. For the single treatment, the reagent was added to each aliquot of 100 μL cell suspension to reach a final PMAxx™ concentration of either 50 μM (2.5 μL of 2 mM PMAxx™) or 100 μM (5 μL of 2 mM PMAxx™). After adding PMAxx™, the HK cell suspensions were incubated in the dark at room temperature for 10 min, followed by exposure to intense light using the PMA Lite™ LED photolysis device (Biotium Inc., Fremont, CA, USA) for 15 min at room temperature. For the sequential treatment, two consecutive treatments of PMAxx™ (2.5 μL of 2 mM PMAxx™ × 2) were added to reach a final concentration of 100 μM, followed by dark and light incubations after each PMAxx™ addition. After the photoactivation(s), PMAxx™-treated HK cell suspensions (both single and sequential treatments) were centrifuged at 17,115× *g* for 5 min and the PBS supernatant with PMAxx™ was removed. Rapid lysis buffer was added to the original tube (where PMAxx™ treatment and photoactivation were carried out) to extract the DNA as described in Section 4.2. Untreated HK cells were run in parallel and all samples were analyzed by qPCR as described in 4.3

#### 4.6.2. Effects of PMAxx™ and Post- PMAxx™ Treatment Conditions on Live Cells

Based on the findings from the experiment above, a 100 μM PMAxx™ concentration was chosen as the PMA treatment. Aliquots of 100 μL of 10^−7^ and 10^−8^ dilutions of untreated and PMAxx™-treated live cells were plated on BAP to determine the PMAxx™ effect on cell viability and growth. 

Serially diluted live cells from neat down to 10^−8^ were treated with a 100 μM final PMAxx™ concentration, and the following three experiments were performed (a) to determine the effect of photoactivation and lysis in the same PMAxx™ treatment tube after photoactivation: PMAxx™ was removed by centrifugation at 17,115× *g* for 5 min and the cells were resuspended in rapid lysis buffer in the same tube. DNA was extracted as described in Section 4.2; (b) determine if a wash step after PMAxx™ treatment could remove residual PMAxx™: after removing PMAxx™ post-photoactivation as in (a), the cell pellet was washed with 1 mL of PBS and was followed by centrifugation at 17,115× *g* for 5 min. The cells were resuspended in 100 μL of rapid lysis buffer in the same tube and DNA was extracted as described in Section 4.2; (c) to determine if a tube change after PMAxx™ treatment could remove residual PMAxx™: after removing PMAxx™ post-photoactivation as in (a), the cell pellet was resuspended in 100 μL of PBS and was transferred to a new tube. The cell suspension was centrifuged in the new tube and the cell pellet was resuspended in 100 μL of rapid lysis buffer for DNA extraction as described in Section 4.2. 

#### 4.6.3. PMAxx™ Attachment to Polypropylene Tube Walls

Findings from Section 4.6.2 led to the hypothesis that PMAxx™ can potentially attach to polypropylene tube walls. To test this hypothesis, PMAxx™ was added to tubes with 100 μL of PBS (without any cells) to a final concentration of 100 μM; the same concentration used for the treatment to coat the insides of the tubes. The PMAxx™+PBS suspension was incubated in the dark for 10 min, photoactivated for 15 min, and then PMAxx™+PBS was removed from each tube. The following was performed (in triplicate): (a) tubes were washed with 1 mL PBS, and then PBS was removed; (b) tubes were not washed. Then, 100 μL of 10^5^ CFU/mL of HK cells in PBS was added to each pre-coated PMAxx™ tube, followed by 10 min dark incubation and 15 min photoactivation. In the same tube, the HK cell suspension was centrifuged at 17,115× *g* for 5 min, the supernatant was removed, and the cell pellet was resuspended in lysis buffer and boiled at 95 °C for 15 min. Ct values from the qPCR assay were compared with 10^5^ CFU/mL HK cells in the absence of PMAxx™ treatment. 

### 4.7. Optimized PMAxx™ Treatment on 100% HK Cells and Live Cells Spiked with Different Concentrations of HK Cells

The final optimized PMAxx™ treatment (100 μM total PMAxx™, photoactivation, removal of PMAxx™ by centrifugation and resuspension in a second set of tubes for extraction) was tested on (a) ten-fold serial dilutions (from 10^8^ to 10^3^) of HK cells and (b) a mixture of different live cell concentrations (neat, 10^−2^, 10^−4^, and 10^−6^) spiked with 10^8^ CFU/mL HK cells at a ratio of 1:1 in a total of 200 μL reaction volume.

### 4.8. Statistical Analysis

Statistical analysis was performed using GraphPad Prism statistical software version 5 (GraphPad Software, Inc., La Jolla, CA, USA). The Student’s *t*-test was used to compare pairs of treatments and analysis of variance (ANOVA) to compare more. Bonferroni post-test was used to analyze treatment differences, and the differences were considered statistically significant when *p* < 0.05.

## 5. Conclusions

In conclusion, our improved vPCR protocol completely removed 10^8^ CFU/mL HK cell signals. In a mixture of live and HK cells, the PMAxx™ treatment eliminated 10^8^ CFU/mL HK cells and selectively amplified live cells. We recommend using a 100 μM PMAxx™ treatment, using separate tubes for the PMAxx™ photoactivation and lysis, and removing PMAxx™ prior to transferring the cells to a new tube for lysis to avoid carryover residual PMA. Further investigations are warranted to improve the sensitivity of the vPCR assay, since even with these considerations, PMAxx™ treatments decreased the sensitivity of the qPCR assay from 10^3^ to 10^4^ CFU/mL. Furthermore, validation using food and clinical specimens is required to improve the vPCR assay and to develop it further as a viability assessment tool.

## Figures and Tables

**Table 1:** Limit of detection for the 14 *Salmonella* serotypes.

Log CFU/mL	Average Ct Value	% Positives
6	20.62 ± 0.31	100.0
5	23.98 ± 0.33	100.0
4	27.22 ± 0.54	100.0
3	30.70 ± 0.62	100.0
2	33.97 ± 1.00	75.6
1	34.55 ± 0.34	2.6

Ct: Cycle threshold. Cell counts in log CFU/mL confirmed by plating. Data were from 2 independent assays performed in triplicates and displayed as mean ± SD.

**Table 2:** Average Ct values for PMAxx™ treatments * on HK cells (without tube change).

Log CFU/mL	HK Untreated	50 μM PMAxx™ × 1 (50 μM Total)	100 μM PMAxx™ × 1 (100 μM Total)	50 μM PMAxx™ × 2 (100 μM Total)
8	14.90 ± 0.68	28.27 ± 0.87	ND	ND
7	18.53 ± 0.32	ND	ND	ND
6	21.75 ± 0.30	ND	ND	ND
5	25.07 ± 0.41	ND	ND	ND

* PMAxx™ treatment (prepared in 20% DMSO): a single treatment of 50 μM PMAxx™ (2.5 μL PMAxx™), a single treatment of 100 μM PMAxx™ (5 μL PMAxx™), and two sequential treatment of 50 μM PMAxx™ (2.5 μL PMAxx™ added twice), where photoactivation step was repeated after each 50 μM PMAxx™ addition; photoactivation and lysis was performed in the same tube (without tube change). Data were from 3 independent assays with triplicates per run and displayed as mean ± SD. CFU: colony-forming unit; HK: heat killed; ND: not detected; PMAxx™: propidium monoazide dye reagent

**Table 3:** Average Ct values for the effect of post-PMAxx™ treatments * under different conditions on live cells.

Log CFU/mL	Untreated without Tube Change ^a^	Untreated with Tube Change ^b^	PMAxx™-Treated without Tube Change ^c^	PMAxx™-Treated with Wash + without Tube Change ^d^	PMAxx™-Treated with Tube Change ^e^	Δ Ct ^c−a^	Δ Ct ^d−a^	Δ Ct ^e−a^
8	14.45 ± 0.23	14.93 ± 0.42	14.34 ± 0.41	15.28 ± 0.54	15.05 ± 0.65	−0.11	0.83	0.60
7	17.70 ± 0.19	17.97 ± 0.15	20.36 ± 1.19	22.02 ± 0.32	18.28 ± 0.27	2.66	4.32	0.58
6	21.07 ± 0.34	21.41 ± 0.38	28.46 ± 0.50	27.25 ± 0.39	22.25 ± 0.81	7.39	6.18	1.18
5	24.06 ± 0.33	24.40 ± 0.23	ND	31.25 ± 0.45	26.61 ± 0.41	NA	7.19	2.55
4	27.29 ± 0.36	27.77 ± 0.27	ND	ND	29.67 ± 0.56	NA	NA	2.38
3	30.99 ± 0.60	31.08 ± 0.29	ND	ND	ND	NA	NA	NA

* PMAxx™ treatment (prepared in 20% DMSO): a 100 μM total PMAxx™, 10 min dark incubation at room temperature, and 15 min exposure to intense light (photoactivation). ΔCt: difference in the cycle threshold; CFU: colony-forming unit; ND: not detected; N/A: not applicable; PMAxx™: propidium monoazide dye reagent. Data were from 3 independent assays with triplicates per run and displayed as mean ± SD.

**Table 4:** Average Ct values for the effect of PMAxx™ treatment * and tube change on HK cells.

Log CFU/mL	HK Untreated without tube Change ^a^	HK Untreated with Tube Change ^b^	HK PMAxx™-Treated without Tube Change	HK PMAxx™-Treated with Tube Change	Δ Ct ^b−a^
8	14.57 ± 0.43	15.98 ± 0.45	ND	ND	1.41
7	17.33 ± 0.18	22.45 ± 0.92	ND	ND	5.12
6	20.63 ± 0.17	25.41 ± 0.42	ND	ND	4.79
5	23.93 ± 0.23	30.19 ± 1.68	ND	ND	6.27
4	27.04 ± 0.24	ND	ND	ND	N/A
3	30.39 ± 0.42	ND	ND	ND	N/A

* With a 100 μM total PMAxx™ concentration, photoactivation and lysis in different tubes (with tube change), and PMAxx™ removed prior to tube change. Data indicate mean ± SD and were from 3 independent assays with triplicates per run. ΔCt: difference in the cycle threshold; ND: not detected.

## Data Availability

Not applicable.

## Supplementary Materials

The following supporting information can be downloaded at: https://www.mdpi.com/article/10.3390/ijms232314708/s1.

## References

[1] Berenger B., Chui L., Reimer A., Allen V., Alexander D., Domingo M.-C., Haldane D., Hoang L., Levett P., MacKeen A. (2017). Canadian Public Health Laboratory Network Position Statement: Non-Culture Based Diagnostics for Gastroenteritis and Implications for Public Health Investigations. Can. Commun. Dis. Rep..

[2] Shea S., Kubota K.A., Maguire H., Gladbach S., Woron A., Atkinson-Dunn R., Couturier M.R., Miller M.B. (2017). Clinical Microbiology Laboratories’ Adoption of Culture-Independent Diagnostic Tests Is a Threat to Foodborne-Disease Surveillance in the United States. J. Clin. Microbiol..

[3] Nogva H.K., Drømtorp S.M., Nissen H., Rudi K. (2003). Ethidium Monoazide for DNA-Based Differentiation of Viable and Dead Bacteria by 5′-Nuclease PCR. Biotechniques.

[4] Nocker A., Camper A.K. (2006). Selective Removal of DNA from Dead Cells of Mixed Bacterial Communities by Use of Ethidium Monoazide. Appl. Environ. Microbiol..

[5] Codony F., Dinh-Thanh M., Agustí G. (2020). Key Factors for Removing Bias in Viability PCR-Based Methods: A Review. Curr. Microbiol..

[6] Fittipaldi M., Nocker A., Codony F. (2012). Progress in Understanding Preferential Detection of Live Cells Using Viability Dyes in Combination with DNA Amplification. J. Microbiol. Methods.

[7] Nocker A., Cheung C.Y., Camper A.K. (2006). Comparison of Propidium Monoazide with Ethidium Monoazide for Differentiation of Live vs. Dead Bacteria by Selective Removal of DNA from Dead Cells. J. Microbiol. Methods.

[8] Codony F., Agustí G., Allué-Guardia A. (2015). Cell Membrane Integrity and Distinguishing between Metabolically Active and Inactive Cells as a Means of Improving Viability PCR. Mol. Cell Probes..

[9] Liu Y., Mustapha A. (2014). Detection of Viable *Escherichia coli* O157: H7 in Ground Beef by Propidium Monoazide Real-Time PCR. Int. J. Food Microbiol..

[10] Agustí G., Fittipaldi M., Codony F. (2017). False-Positive Viability PCR Results: An Association with Microtubes. Curr. Microbiol..

[11] Desneux J., Chemaly M., Pourcher A.M. (2015). Experimental Design for the Optimization of Propidium Monoazide Treatment to Quantify Viable and Non-Viable Bacteria in Piggery Effluents. BMC Microbiol..

[12] Kralik P., Nocker A., Pavlik I. (2010). *Mycobacterium avium* Subsp. Paratuberculosis Viability Determination Using F57 Quantitative PCR in Combination with Propidium Monoazide Treatment. Int. J. Food Microbiol..

[13] van Holm W., Ghesquière J., Boon N., Verspecht T., Bernaerts K., Zayed N., Chatzigiannidou I., Teughels W. (2021). A Viability Quantitative PCR Dilemma: Are Longer Amplicons Better?. Appl. Environ. Microbiol..

[14] Government of Canada (2020). National Enteric Surveillance Program (NESP) Annual Summary 2019.

[15] Public Health Agency of Canada (2016). Food-Related Illnesses, Hospitalizations, & Deaths in Canada. https://www.canada.ca/en/public-health/services/publications/food-nutrition/infographic-food-related-illnesses-hospitalizations-deaths-in-canada.html.

[16] Government of Canada (2015). National Enteric Surveillance Program (NESP) Annual Report 2012.

[17] Galan J.E., Curtiss R. (1991). Distribution of the InvA, -B, -C, and -D Genes of *Salmonella* Typhimurium among Other *Salmonella* Serovars: *InvA* Mutants of *Salmonella* Typhi Are Deficient for Entry into Mammalian Cells. Infect. Immun..

[18] Zeng D., Chen Z., Jiang Y., Xue F., Li B. (2016). Advances and Challenges in Viability Detection of Foodborne Pathogens. Front Microbiol..

[19] Kellner T., Parsons B., Chui L., Berenger B.M., Xie J., Burnham C.A.D., Tarr P.I., Lee B.E., Nettel-Aguirre A., Szelewicki J. (2019). Comparative Evaluation of Enteric Bacterial Culture and a Molecular Multiplex Syndromic Panel in Children with Acute Gastroenteritis. J. Clin. Microbiol..

[20] Nocker A., Sossa K.E., Camper A.K. (2007). Molecular Monitoring of Disinfection Efficacy Using Propidium Monoazide in Combination with Quantitative PCR. J. Microbiol. Methods.

[21] Taylor M.J., Bentham R.H., Ross K.E. (2014). Limitations of Using Propidium Monoazide with QPCR to Discriminate between Live and Dead *Legionella* in Biofilm Samples. Microbiol. Insights.

[22] Takahashi H., Gao Y., Miya S., Kuda T., Kimura B. (2017). Discrimination of Live and Dead Cells of *Escherichia coli* Using Propidium Monoazide after Sodium Dodecyl Sulfate Treatment. Food Control.

[23] Barbau-Piednoir E., Mahillon J., Pillyser J., Coucke W., Roosens N.H., Botteldoorn N. (2014). Evaluation of Viability-QPCR Detection System on Viable and Dead *Salmonella* Serovar Enteritidis. J. Microbiol. Methods.

[24] Kobayashi H., Oethinger M., Tuohy M.J., Hall G.S., Bauer T.W. (2009). Improving Clinical Significance of PCR: Use of Propidium Monoazide to Distinguish Viable from Dead *Staphylococcus aureus* and *Staphylococcus epidermidis*. J. Orthop. Res..

[25] Thanh M.D., Agustí G., Mader A., Appel B., Codony F. (2017). Improved Sample Treatment Protocol for Accurate Detection of Live *Salmonella* Spp. in Food Samples by Viability PCR. PLoS ONE.

[26] Geniul F.C. (2016). Understanding the Tube Contribution in VPCR Results Understanding the Tube Contribution in VPCR Results. Res. Publ..

[27] Halder S., Yadav K.K., Sarkar R., Mukherjee S., Saha P., Haldar S., Karmakar S., Sen T. (2015). Alteration of Zeta Potential and Membrane Permeability in Bacteria: A Study with Cationic Agents. Springerplus.

[28] Smith D.E., Dhinojwala A., Moore F.B.G. (2019). Effect of Substrate and Bacterial Zeta Potential on Adhesion of *Mycobacterium smegmatis*. Langmuir.

[29] Schmidlin M., Alt M., Brodmann P., Bagutti C. (2010). Insufficient Distinction between DNA from Viable and Nonviable *Staphylococcus aureus* Cells in Wipe-Samples by Use of Propidium Monoazide-PCR. Appl. Biosaf..

[30] Wu B., Liang W., Kan B. (2015). Enumeration of Viable Non-Culturable *Vibrio cholerae* Using Propidium Monoazide Combined with Quantitative PCR. J. Microbiol. Methods.

