# Supplementary material for: An Improved Real-Time Viability PCR Assay to Detect Salmonella in a Culture-Independent Era
Source: Int J Mol Sci. 2022 Nov 25;23(23):14708. doi: 10.3390/ijms232314708 (PMC9738789; doi:10.3390/ijms232314708)
Supplement: Supplementary file 1 [file ijms-23-14708-s001.zip › ijms-2045620-supplementary.pdf]

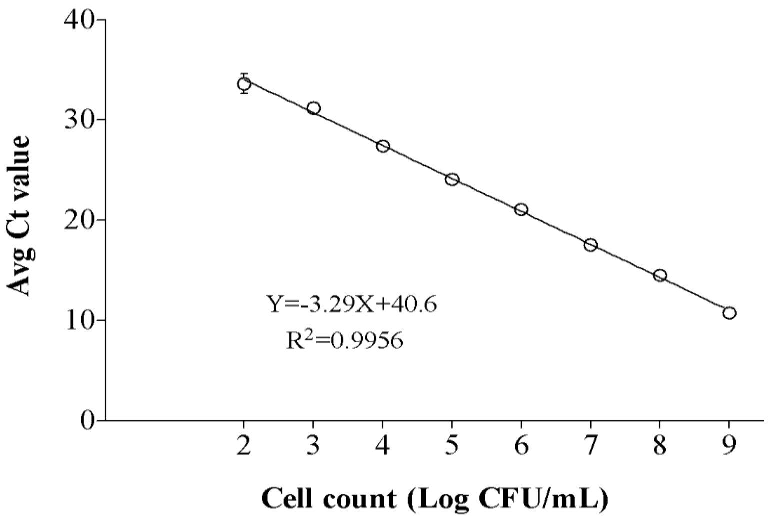

Supplementary Figure S1: Standard curve showing the correlation of average Ct values and cell counts from *Salmonella* Enteritidis. Data were from 3 independent assays with triplicates per qPCR run and plate growth and displayed as mean ± SD. Ct: cycle threshold, CFU: colony-forming units.

Supplementary Table S1: Sequence of the in-house primers and the probe targeting the *invA* gene

	Sequence (5′-3′)	Reference
Forward	CTGCGGTACTGTTAATTAC	[13]
Reverse	GAACGTGGCGATAATTTC	
Probe	6-FAM-CGGCATCGG/ZEN/CTTCAATCAAGA-IowaBlack® FQ	

The product length: 104 bp (BLASTN, NIH).

Supplementary Table S2: The *Salmonella* serotypes used for inclusivity and non-*Salmonella* strains used for exclusivity assays

***Salmonella* serotypes (n=14)**	**Non-*Salmonella* strains (n=17)**
S. enterica subsp. *enterica* ser. Farmsen	*Aeromonas hydrophila* (Clinical strain)
S. enterica subsp. *enterica* (I) ser. 4,[5], 12:i:-	*Bacillus cereus* (ATCC14579)
S. enterica subsp. *enterica* ser. Infantis	*Citrobacter freundii* (ATCC8090)
S. enterica subsp. *enterica* ser. Thompson	*Enterobacter aerogenes* (ATCC13048)
S. enterica subsp. *enterica* ser. Sundsvall	*Enterobacter cloacae* (ATCC13047)
S. enterica subsp. *enterica* ser. Kiambu	*Escherichia coli O157:H7* (ATCC35150)
S. enterica subsp. *enterica* ser. Goldcoast	*Escherichia coli* (ATCC25922)
S. enterica subsp. *enterica* ser. Heidelberg	*Klebsiella pneumonia* (ATCC31488)
S. enterica subsp. *enterica* ser. Reading	*Proteus mirabilis* (ATCC12453)
S. enterica subsp. *enterica* ser. Offa	*Pseudomonas aeruginosa* (ATCC27853)
S. enterica subsp. *enterica* ser. Lome	*Shigella boydii* (Clinical strain)
S. enterica subsp. *enterica* ser. Newport	*Shigella dysenteriae* (Clinical strain)
S. enterica subsp. *enterica* ser. Typhimurium	*Shigella flexneri* (Clinical strain)
S. enterica subsp. *enterica* ser. Enteritidis	*Shigella sonnei* (Clinical strain)
	Staphylococcus aureus (ATCC25913)
	Staphylococcus epidermidis (ATCC12228)
	Yersinia enterocolitica (Clinical strain)

Subsp.: subspecies; ser.: serotype

Supplementary Table S3: Effect of optimized PMAxx™ treatment* on 10^8 CFU/mL HK cells spiked into live cell dilutions from 10^9 to 10^3 CFU/mL (Average Ct values used for Figure 2).

Live cell dilution (Log CFU/mL)	HK cell dilution (Log CFU/mL)	Average Ct values					
		HK untreated	HK- PMAxx™ treated	Live-untreated	Live- PMAxx™ treated	Live+HK-untreated	Live+HK-PMAxx™ treated
None	8	15.34 ± 0.15	31.06 ±0.68	-	-	-	-
9	None	-	-	10.50 ± 0.32	10.37 ± 0.07	-	-
7	None	-	-	17.31 ± 0.03	17.65 ± 0.08	-	-
5	None	-	-	23.64 ± 0.09	25.77 ± 0.20	-	-
3	None	-	-	31.11 ± 0.49	ND	-	-
9	8	-	-	-	-	11.19 ± 0.19	11.36 ± 0.12
7	8	-	-	-	-	16.65 ± 0.13	19.05 ± 0.14
5	8	-	-	-	-	17.25 ± 0.07	26.34 ± 0.34
3	8	-	-	-	-	17.11± 0.10	ND

* With a 100 μM total PMAxx™ concentration, photoactivation and lysis in different tubes (with tube change), and PMAxx™ removed prior to tube change. Data were from 3 independent assays with triplicate qPCR wells and displayed as mean ± SD. HK: Heat-killed, ND: not detected
